# Blood Eosinophil Endotypes across Asthma and Chronic Obstructive Pulmonary Disease (COPD)

**DOI:** 10.1155/2022/9656278

**Published:** 2022-10-19

**Authors:** Jia-Ying Luo, Hui-An Chen, Yuan-Yu Feng, Ye-Peng Chen, Xiang-Cheng Lei, Shuang-Lin Guo, Xiao-Bin Huang, Zhi-Man Liang, Ning Li, Bao-Qing Sun

**Affiliations:** ^1^Department of Allergy and Clinical Immunolog, Department of Laboratory, National Center for Respiratory Medicine, National Clinical Research Center for Respiratory Disease, State Key Laboratory of Respiratory Disease, Guangzhou Institute of Respiratory Health, The First Affiliated Hospital of Guangzhou Medical University, Guangdong, China; ^2^Guangzhou Medical University, Guangzhou, Guangdong, China

## Abstract

**Background:**

Eosinophils were common inflammatory cells involved in the occurrence and development of various inflammatory diseases. Multiple recent studies have pointed to the increasingly important role of eosinophils in respiratory diseases. This article aims to compare the expression differences of blood eosinophil counts between asthma, chronic obstructive pulmonary disease (COPD), and asthma-COPD overlap (ACO).

**Methods:**

Patients with asthma, COPD, and ACO who were seen in the First Affiliated Hospital of Guangzhou Medical University from January 2012 to June 2019 were included. We collected information such as age, gender, diagnosis, the eosinophil counts from the medical records. Moreover, the levels of 10 cytokines in the plasma of each group were detected by using the Meso Scale Discovery method.

**Results:**

We included 9787 patients with asthma, 15806 patients with COPD, and 831 ACO patients. From our results, it can be first found that eosinophil levels were age-related in the three diseases (asthma and ACO: *p* < 0.001; COPD: *P* = 0.001); in asthma and COPD, the number of eosinophils in males was more significant than that in females (asthma: *p* < 0.001; COPD: *p* = 0.012). Second, asthma patients had higher blood eosinophil counts than those with COPD and ACO (*p* < 0.001). Moreover, we found out that eosinophil levels were highly expressed in the stable group of all three diseases. Finally, we found that most cytokines in ACO patients showed a downward trend when the level of eosinophils was low, whereas the results were reversed in asthma patients; 7 cytokines had similar trends in COPD and ACO patients.

**Conclusions:**

In conclusion, eosinophils have their own unique endotypes in asthma, COPD, and ACO patients, which were reflected in the fluctuation of their levels and changes in cytokine secretion.

## 1. Introduction

Asthma and chronic obstructive pulmonary disease (COPD) are common chronic respiratory diseases, and the prevalence of both diseases increases yearly worldwide [1]. Moreover, COPD has been considered the third most common cause of death in 2020 [2]. Research in recent years found that not only asthma but also COPD has multiple endotypes, which is reflected in the differences in airway inflammation characteristics [3–5]. The differences in the manifestations of inflammation between individuals will impose additional burdens on diagnosis and treatment. Thus, identifying their various endotypes plays an important role in guiding clinical treatment.

Eosinophils are important inflammatory cells in the body. They actively promote various inflammatory cascades by producing and releasing chemokines or cytokines [6]. Eosinophilia is a common manifestation of asthma, and a study has shown that half of asthmatics have eosinophilia [7]. Environmental pollutants or allergens such as pollen affect the release of eosinophils, which illustrates that eosinophils can also be used to assess the severity of asthma.

COPD is characterized by airway obstruction that is not completely reversible. Studies have found that the incidence of eosinophilic inflammation in COPD patients may be as high as 45% [8]. In COPD, not only is eosinophilic inflammation suggested for COPD exacerbation but eosinophilia is associated with the ACO endotype, which is associated with more wheezing [9–11]. Furthermore, the blood eosinophil counts of COPD patients can be used as a predictive biomarker of response to inhaled or oral corticosteroids [12]. All of the above findings suggest that eosinophils may play a role in COPD. In addition, approximately 20% of patients with obstructive airway disease have asthma and COPD features, called asthma-COPD overlap (ACO) [13]. Compared with COPD, this type of patient has a more pronounced airway morphological change, milder change in emphysema, and a more significant increase in eosinophil levels [14, 15].

It is the common method for clinicians to diagnose asthma and COPD by combining family medical histories and clinical characteristics, supplemented by various diagnostic methods such as lung function and bronchial provocation testing [16]. Recently, some studies have pointed out that the levels of eosinophils in the blood may have an association with those in the lung and can hence reflect eosinophilic inflammation in the airways, improving the efficiency of clinical inspection [17, 18]. Therefore, in this study, we used eosinophils as the main line to compare their differential expressions in asthma, COPD, and ACO patients and explore the distributions of eosinophils in stable and acute patients. We aimed to analyze the different expression patterns of eosinophils, understanding the expression differences of eosinophil counts between asthma, COPD, and ACO.

## 2. Materials and Methods

### 2.1. Study Design

This study is a retrospective analysis aimed at distinguishing the eosinophilic endotypes in asthma, COPD, and ACO using levels of inflammatory cells and cytokines. We compared eosinophil and neutrophil counts in asthma, COPD, and ACO patients; contrasted the diversification of eosinophil and neutrophil between the acute and the stable groups. The cutoff levels of 0.3 × 10^9^/L for eosinophils and 7 × 10^9^/L for neutrophils were chosen as representative upper reference levels (upper limit of normal). To further evaluate the mechanism of eosinophil proliferation and expression differences in asthma, COPD, and ACO, we selected pretreatment blood samples for cytokine analysis. Plasma was collected within one week before the measurement of cytokines, excluding a few sample volumes, hemolysis, and cloudy or milky plasma. The ethics committee approved the study from the First Affiliated Hospital of Guangzhou Medical University (GYFYY-2016-73), and individual consent for this retrospective analysis was waived.

### 2.2. Participants

The current study selected 26424 adults, including outpatient and inpatient, from the First Affiliated Hospital of Guangzhou Medical University between January 2012 and June 2019. We collected basic information such as the patient's age, gender, and diagnosis through medical records. The diagnostic basis and inclusion and exclusion criteria of each group were as follows: According to the 2011 GINA diagnostic guidelines, 9787 patients in the asthma group had different degrees of respiratory symptoms, and FEV1 increased by more than 12% after using bronchodilators [19]. Based on the symptoms, 8708 patients were divided into the stable group and 1079 into the acute group. Patients in the asthma group were not diagnosed with COPD by pulmonary function detection. There were 15806 inpatients diagnosed with COPD based on the Global Initiative for Chronic Obstructive Lung Disease (GOLD) guideline in 2011 [20]. They had clinical symptoms such as dyspnea and chronic or sputum production. FEV1/FVC was lower than 0.7 after using bronchodilators, which confirmed the presence of persistent airflow limitation. Moreover, none of the patients in this group met the diagnostic criteria of asthma. On the basics of COPD, 12505 patients presented with at least two main symptoms (increased dyspnea, increased sputum purulent or increased sputum volume) or one main symptom and one secondary symptom (nasal discharge/congestion, wheezing, sore throat or cough) for at least two consecutive days were subdivided as acute COPD. The remaining 3301 patients were classified as stable COPD. Finally, 831 inpatients who met the diagnostic criteria for asthma and COPD groups were classified as ACO group, including 190 with stable and 641 with acute. Patients in this group had clinical manifestations of asthma and symptoms such as COPD airway obstruction and met pulmonary function indicators. The outpatient patients were followed up regularly in outpatient clinics, with the symptoms stable for at least one month, and the hospitalized patients were in acute exacerbation. All the patients in this study were excluded from lung-related complications, such as bronchiectasis, lung cancer, and severe heart, liver, kidney, or other systemic diseases.

### 2.3. Eosinophil, Neutrophil, and Cytokine Testing

We obtained eosinophil and neutrophil counts from each participant's routine blood tests in their medical records. To further understand the influence of eosinophils in asthma, COPD, and ACO, we selected pretreatment blood samples for cytokine analysis. To ensure the accuracy of the results, the plasma was collected within one week before the cytokine detection for the experiment. The Meso Scale Discovery (MSD) method was used to analyze the levels of ten cytokines at different eosinophil levels in each patient. The ten cytokines tested included IFN-*γ*, IL-1*β*, IL-2, IL-4, IL-6, IL-8, IL-10, IL-12p70, IL-13, and TNF-*α*. For plasma collection, we took 3–5 ml of peripheral blood, which would be centrifuged at 3000 rpm at room temperature for 5 minutes, and then collected the plasma and stored it at −80°C. Considering the problems of sample volume, hemolysis, and cloudy or milky plasma, we excluded 16 samples that could not be used in this experiment. Finally, only 30 patients in each group were left for cytokine detection. 96-well plates filled with different antibodies and plasma were then energized to obtain the cytokine levels of the 90 patients.

### 2.4. Statistical Analysis

All data were analyzed by using the Statistical Package for the Social Science software 24.0 (SPSS, IBM Corp, Armonk, NY, USA). The numerical values in this study were presented as the mean ± standard deviation (SD). We used Dunnett's test to analyze the correlation between the three groups and the two types of blood cells. Pearson correlation coefficient was used to evaluate whether gender or age correlated with eosinophil or neutrophil levels in the three groups. Finally, the correlation of the ten cytokines with different levels of eosinophils in the three groups was determined by using the Mann-Whitney *U* test. *p* values <0.05 were considered statistically significant. All figures in this study were drafted by using GraphPad Prism 8.

## 3. Results

### 3.1. The Relationship between Blood Cell Levels and Gender or Age in the Three Groups

**Figure 1:**
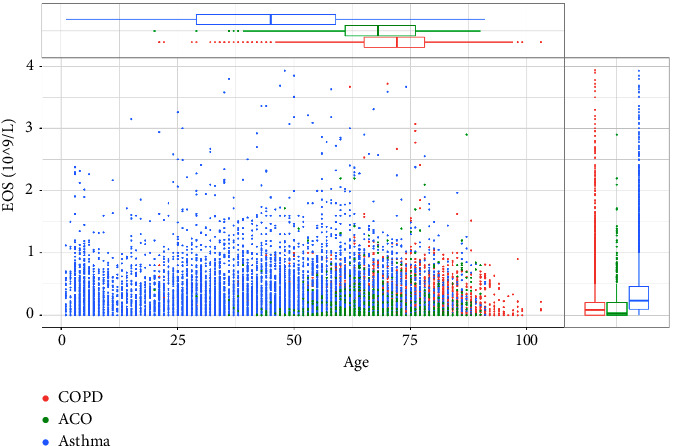
Distribution of eosinophils at different ages in the three diseases. (COPD, chronic obstructive pulmonary disease; ACO, asthma-COPD overlap; EOS, eosinophils).

**Figure 2:**
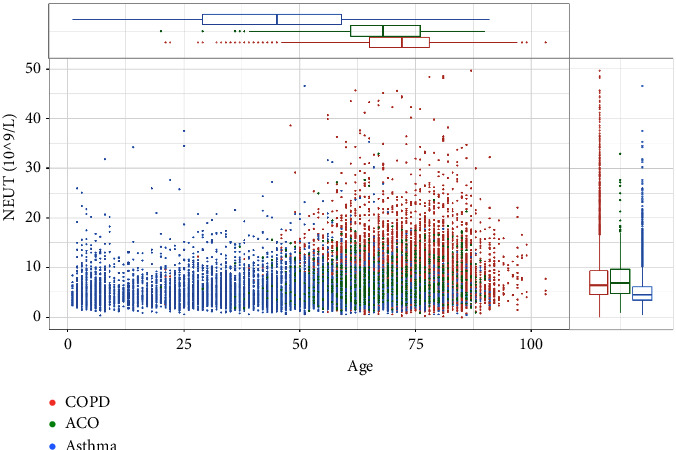
Distribution of neutrophils at different ages in the three diseases. (COPD, chronic obstructive pulmonary disease; ACO, asthma-COPD overlap; NEUT, neutrophils).

In this study, the demographics of asthma, COPD, and ACO were summarized in Table 1. We analyzed the relationship between the three groups and demographics, such as age and sex, to determine whether the blood cell counts varied per group. In the asthma group, we found that both eosinophils and neutrophils had a significant relationship with age. However, eosinophils correlated negatively, while neutrophils correlated positively with age (eosinophils: *r* = −0.082, *p* < 0.001; neutrophils: *r* = 0.117, *p* < 0.001). Moreover, we discovered that the ACO group also had a similar relationship (eosinophils: *r* = −0.147, *p* < 0.001; neutrophils: *r* = 0.08, *p*=0.019). In contrast to the other groups, in the COPD group, we found that both eosinophil and neutrophil blood levels negatively correlated with age (eosinophils: *r* = −0.03, *p*=0.001; neutrophils: *r* = −0.02, *p*=0.025). The distributions of eosinophils and neutrophils at different ages are shown in Figures 1 and 2.

**Figure 3:**
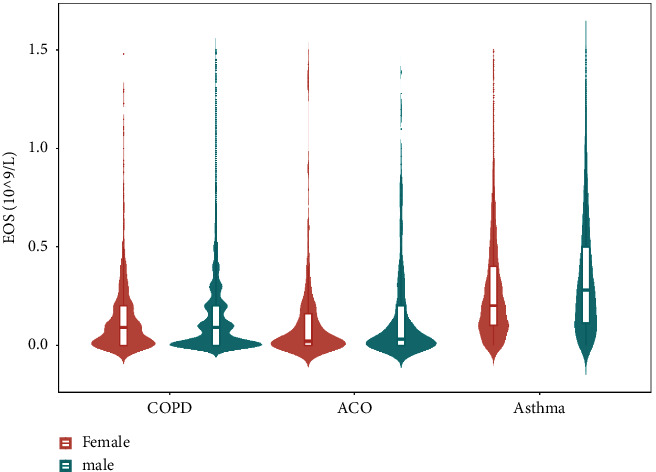
Distribution of eosinophils by gender in the three diseases. (COPD, chronic obstructive pulmonary disease; ACO, asthma-COPD overlap; EOS, eosinophils).

There are many clinical indicators that vary slightly with gender, and the detection levels of each index are no exception. We compared the correlation between the eosinophil levels in the three groups with gender. For asthma and COPD groups, there was a significant correlation between eosinophil counts and sex (asthma: *p* < 0.001; COPD: *p*=0.012). Further, we found out that the average levels of eosinophils in males were higher than those in females. In contrast, there was no obvious correlation in the ACO group (*p*=0.534). Eosinophil levels in males and females are presented in Figure 3.

### 3.2. Comparison of Eosinophil and Neutrophil Counts in the Three Groups

**Figure 4:**
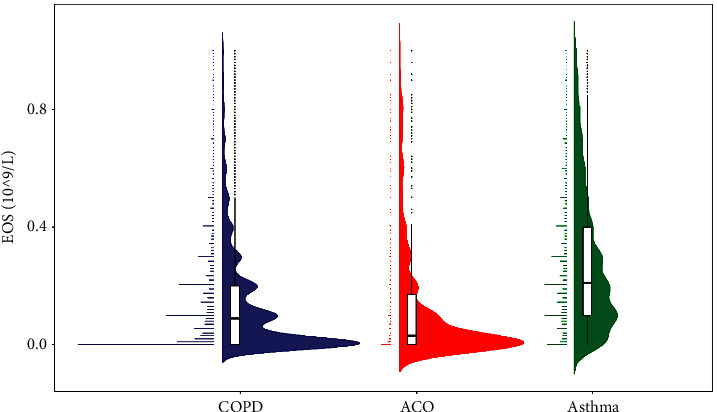
The eosinophil counts in the three study populations. (COPD, chronic obstructive pulmonary disease; ACO, asthma-COPD overlap; EOS, eosinophils).

**Figure 5:**
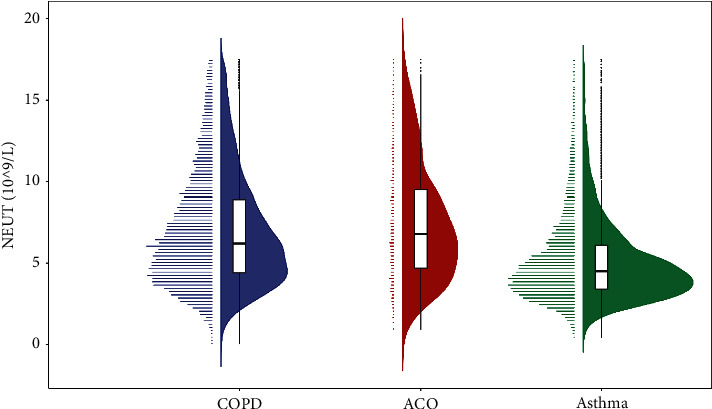
The neutrophil counts in the three study populations. (COPD, chronic obstructive pulmonary disease; ACO, asthma-COPD overlap; NEUT, neutrophils).

On comparing the eosinophil counts of the three groups, we found that the asthma group had significantly higher values than those of the COPD and ACO groups (both *p* < 0.001). However, we did not find any obvious differences between the COPD and ACO groups (*p*=0.995). Interestingly, when we compared the neutrophil counts, we found an opposite trend in the results. The neutrophil counts in the COPD and ACO groups were significantly higher than those in the asthma group (*p*=0.001 and *p* < 0.001, respectively). However, the values for the COPD and ACO groups were not statistically different (*p*=0.948). The distribution patterns of the two blood cells in the three groups are presented in Figures 4 and 5.

### 3.3. The Magnitude of Changes in Eosinophils and Neutrophils between Stable and Acute Patients

**Figure 6:**
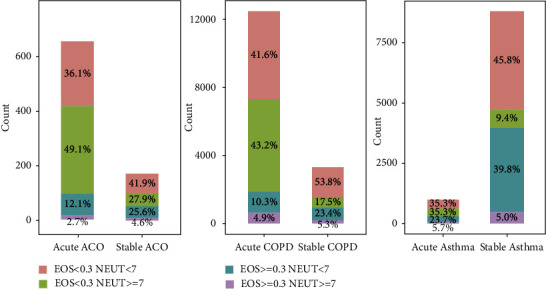
Population distribution of eosinophil and neutrophil levels in acute, or stable groups and their proportions at each level in asthma, COPD or ACO patients. We differentiated acute and stable patients in asthma, COPD, and ACO groups according to eosinophils 0.3 × 10^9^/L and neutrophils 7 × 10^9^/L, respectively. The percentages showed the proportions of patients with different cell levels combinations in the total number of stable or acute patients, where blue and purple presented the percentage of eosinophils greater than or equal to 0.3 × 10^9^/L; red and green presented the percentage of eosinophils less than 0.3 × 10^9^/L; green and purple were the percentage of neutrophil levels greater than or equal to 7 × 10^9^/L; red and blue were the percentage of neutrophil levels less than 7 × 10^9^/L. The column height corresponds to the number of people in each group on the *Y* axis. (COPD, chronic obstructive pulmonary disease; ACO, asthma-COPD overlap; EOS, eosinophils; NEUT, neutrophils).

Comparing the distribution of eosinophils in patients with stable and acute diseases (Figure 6), we found that 44.8% of patients with stable asthma had eosinophil levels above 0.3 (10^9^/L). This distribution was much higher than 29.4% of acute asthma patients. In contrast, the population with neutrophil levels higher than 7 (10^9^/L) were mainly acute patients and higher than those in stable patients (41.0% and 14.4%).

We obtained similar results after comparing the cellular levels of patients in the COPD and ACO groups, respectively. Regardless of COPD or ACO patients, nearly half of patients in the acute group had neutrophil levels greater than 7 (10^9^/L) (COPD: 48.1% and ACO: 51.8%), which was higher than the percentage of high eosinophils (COPD: 15.2% and ACO: 14.8%). Moreover, in both COPD and ACO, there was no difference in the proportions of eosinophils above 0.3 (10^9^/L) and neutrophils greater than 7 (10^9^/L) in the stable group. The ratio of COPD patients with high levels of eosinophil or neutrophil was 28.7% and 22.8%, respectively, and ACO was 30.2% and 32.5%.

### 3.4. Correlation between the Ten Cytokines and the Three Diseases with Different Levels of Eosinophils

**Figure 7:**
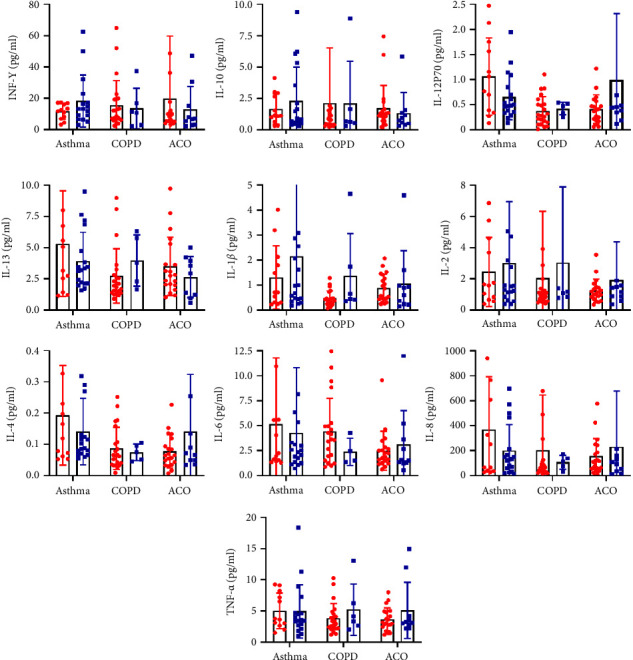
The levels of ten cytokines in the three diseases at different eosinophil levels. Red: eosinophil levels <0.3 (10^9^/L), Blue: eosinophil levels ≧ 0.3 (10^9^/L). All data are mean ± SD of 6 to 24 samples from each group. ^*∗*^*p* < 0.05. (COPD, chronic obstructive pulmonary disease; ACO, asthma-COPD overlap; SD: standard deviation).

Inflammation is a major factor among these three diseases, so we further performed cytokine analysis in each group. The characteristics of patients from each group were summarized in Table 2. We analyzed the changing trends of 10 cytokines at a 0.3 (10^9^/L) cutoff of eosinophils. The number of samples with eosinophils greater than or equal to 0.3 (10^9^/L) and less than 0.3 (10^9^/L) was 18 and 12 in the asthma group; the COPD group was 6 and 24, respectively; the ACO group was 10 and 20, respectively. Interestingly, we found that most of the cytokines (IL-12p70, IL-13, IL-4, IL-6, TNF-*α*, and IL-8) in the asthma patients tend to increase when the low expression of eosinophils; but in the ACO group, only INF-*γ* and IL-10 showed an increase. Moreover, except for IL-2, IL-6, and TNF-*α*, the COPD and ACO group had the same cytokine changed trend: when the expression of eosinophils was low, the secretion of INF-*γ* and IL-10 were increased; IL-12p70, IL-13, IL-1*β*, IL-4, and IL-8 were decreased. However, only IL-1*β* and IL-2 had the same changing trend between asthma and ACO groups. The levels of the ten cytokines in the three groups are shown in Figure 7.

## 4. Discussion

Asthma, COPD, and ACO are increasingly common diseases that endanger our health. The prevalence of asthma is much higher in children and teenagers, but COPD and ACO are more common in adults, especially above 40 years old [21]. Not only is eosinophilic inflammation a characteristic feature of asthma, but in recent years, eosinophils are also recommended for therapeutic decisions in COPD. In our results, eosinophil levels had a low correlation with age in all three diseases. In addition, in asthma and COPD groups, the average levels of eosinophil in males were higher than those in females. This indicated that age and gender might also be factors that affected the levels of eosinophils.

In our study, we compared eosinophils in the three groups. The results showed that the levels of eosinophils in asthma patients were significantly higher than those in COPD and ACO patients (both *p* < 0.001), but no significant differences were found between COPD and ACO. This indicated that asthma and COPD were dominated by different types of inflammation. In addition, although the eosinophil level of ACO was only slightly higher than that of COPD patients, this may indicate that ACO had a more pronounced effect on eosinophils under the action of asthma. Therefore, we speculated that on the basis of asthma, eosinophilic expression was more prominent in ACO than in COPD.

Whether eosinophils can predict the risk of disease deterioration is still controversial [22, 23]. A previous study has shown that patients with acute aggravation of COPD show high-level eosinophils at admission and are associated with a higher risk of moderate-to-severe exacerbations in the 12-month follow-up [24]. Still, there was an inverse correlation between C-reactive protein and eosinophil count in a real-world five-year hospitalization cohort [25], and the proportion of high-level eosinophils was low (15%), which was similar to our results (15.2%); combined with neutrophils, we found that in the low levels of eosinophils group, the proportion change in neutrophils was huge (43.2% vs. 17.5%) compared with the stability stage, causing the deterioration of disease due to infection in most patients. These results indicated eosinophils are not directly related to patients with exacerbations but can reflect the patient's lung function [26]; in our study, it was found that some patients with stable COPD also maintained high levels of eosinophils and whether these patients tended to exacerbations or not needs to be deeper investing. Thus, eosinophil count could help to predict outcomes. A study has shown that severe and stable asthma, COPD, and ACO patients are expected in eosinophilic inflammation [27]. This may indicate that eosinophils still participate in developing the three diseases and play a role. We further compared the change range of eosinophils and neutrophils, finding out that the two cells' change ranges did not match. This showed that there were other factors that caused the changes in eosinophils and neutrophils to be inconsistent. For this result, we wondered about the changes in the organism's immune response in different inflammatory states. In order to explore our doubts, we focused on acute patients to analyze the expression of cytokines.

Cytokines are secreted proteins involved in growth, differentiation, and activation [28]. For example, IL-4 is a highly active cytokine that can influence T-cell differentiation; IL-6 is considered a proinflammatory cytokine-induced by lipopolysaccharide, along with TNF-a and IL-1, and it has both proinflammatory and anti-inflammatory properties [29]. There was a study proposing to use of eosinophils level as an auxiliary means to evaluate the effectiveness of patients with inflammation treatment [12]. Therefore, we divided each of the 30 acute patients into two groups according to the cutoff value of eosinophil 0.3 (10^9^/L) and compared the trend of the changes in cytokines at different levels of eosinophils. We found that at low levels of eosinophils, the secretion of cytokines such as L-12p70, IL-13, and IL-4 in patients with asthma increased, while in the ACO group, the opposite result was shown that most cytokines had a decreasing trend. Furthermore, seven cytokines, including INF-*γ*, IL-10, IL-12p70, and IL-13, showed the same trend in COPD and ACO groups. Some studies put forward that the levels of IL-8 in asthma were lower than those in COPD [30, 31]. Meanwhile, IL-4 and TNF-*α* values in both COPD and ACO were significantly higher than those in asthma [31]. In summary, cytokines expression varied among diseases due to the different disease mechanisms, but although the expression levels were different, there still had a similar trend between COPD and ACO.

Nevertheless, our study still has some limitations. Eosinophil counts were gathered for this research between 2012 and 2019. However, the 2021 strategic report of the Global COPD Initiative proposed that the term asthma-COPD overlap was no longer used due to the difficulty of diagnosing. Instead, the emphasis was on the individualized treatment of patients with asthma or nonCOPD. Therefore, this study can only represent the results of the previous disease. Due to the lack of pre-enrollment treatment history, body mass index, smoking history, and so on, we could not rule out the possibility that other confounding factors may affect eosinophil levels. In addition, there were less research on the different comparisons based on different levels of eosinophil, and we only analyzed the cytokines in this study. Finally, the kinds of cytokines and sample numbers were restricted, which let us not find significant differences between the different EOS levels in the three diseases.

## 5. Conclusion

In conclusion, eosinophils have their own unique endotypes in asthma, COPD, and ACO patients, which were reflected in the fluctuation of their levels and changes in cytokine secretion. The mechanism of action of eosinophils in these diseases remains the main direction of future research.

## Figures and Tables

**Table 1:** Baseline characteristics of participants (COPD: chronic obstructive pulmonary disease; ACO: asthma-COPD overlap; SD: standard deviation).

Parameter	Asthma	COPD	ACO
Total number	9787	15806	831
Male (female) number	4300 (5487)	14247 (1559)	617 (214)
Age (mean ± SD)	42.7 ± 20.6	70.8 ± 9.8	67.9 ± 10.8
Male	39.1 ± 21.4	70.4 ± 9.5	66.3 ± 9.9
Female	45.5 ± 19.5	74.1 ± 11.4	72.8 ± 12.0
Eosinophils (10^9^/L)	0.356 ± 0.525	0.165 ± 0.294	0.164 ± 0.370
Male	0.406 ± 0.588	0.167 ± 0.300	0.168 ± 0.314
Female	0.317 ± 0.465	0.147 ± 0.239	0.150 ± 0.498
Neutrophils (10^9^/L)	5.181 ± 2.893	7.568 ± 4.803	7.641 ± 4.017
Male	5.244 ± 2.974	7.650 ± 4.845	7.793 ± 4.068
Female	5.131 ± 2.828	6.817 ± 4.331	7.202 ± 3.840

**Table 2:** Baseline characteristics of 30 participants from three groups (COPD, chronic obstructive pulmonary disease; ACO, asthma-COPD overlap; SD, standard deviation).

Parameter	Asthma	COPD	ACO
Total number	30	30	30
Male (female) number	10 (20)	27 (3)	24 (6)
Age (mean ± SD)	47.2 ± 15.1	71.5 ± 8.5	67.1 ± 10.0
Male	40.3 ± 19.2	70.4 ± 7.9	65.0 ± 8.4
Female	50.7 ± 11.7	81.0 ± 9.8	75.3 ± 12.4
Eosinophils (10^9^/L)	0.41 ± 0.41	0.17 ± 0.20	0.20 ± 0.29
Male	0.36 ± 0.39	0.18 ± 0.21	0.19 ± 0.31
Female	0.44 ± 0.42	0.10 ± 0.00	0.24 ± 0.17
Neutrophils (10^9^/L)	6.45 ± 3.37	7.21 ± 3.98	7.03 ± 3.02
Male	7.37 ± 3.20	7.01 ± 3.93	7.27 ± 3.21
Female	5.99 ± 3.44	9.03 ± 4.76	6.09 ± 2.06

## Data Availability

The datasets used and/or analyzed during the current study are available from the corresponding author upon reasonable request.

## Abbreviations

COPD: Chronic obstructive pulmonary disease
ACO: Asthma-COPD overlap
GINA: The global initiative for asthma
GOLD: Global initiative for chronic obstructive lung disease
MSD: Meso scale discovery
SD: Standard deviation.
